# Supplementary material for: Connexin-46 Contained in Extracellular Vesicles Enhance Malignancy Features in Breast Cancer Cells
Source: Biomolecules. 2020 Apr 28;10(5):676. doi: 10.3390/biom10050676 (PMC7277863; doi:10.3390/biom10050676)
Supplement: Supplementary file 1 [file biomolecules-10-00676-s001.pdf]

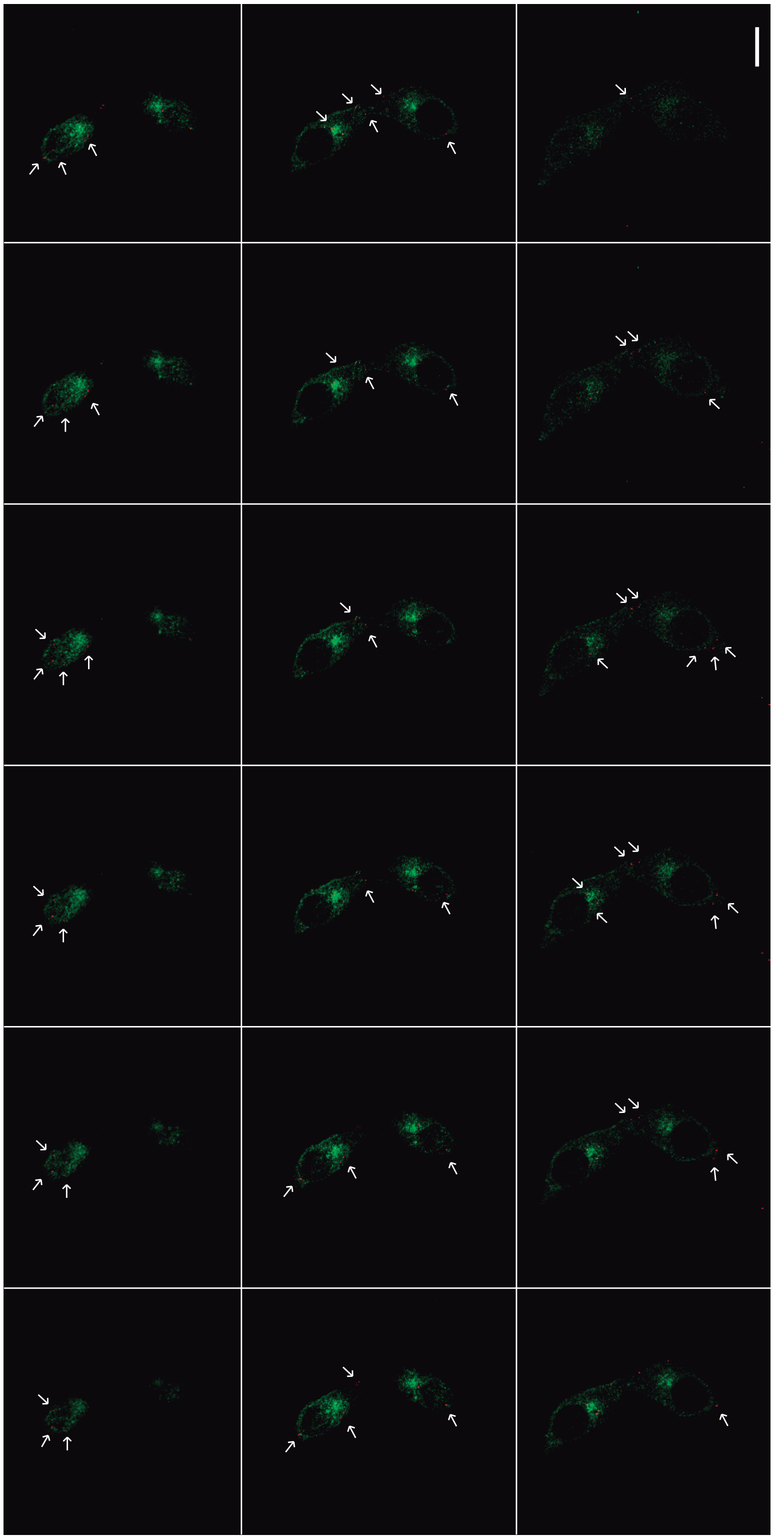

Supplementary figure 1. Confocal microscope analysis of EVs-Cx46 internalization in MCF-7Cx46-GFP and MCF-7 cells at 3 hours. Z-stack images were collected at 0,5 µm intervals. CX46-GFP (green), EVs-Cx46 (red). White arrows indicate the EVs-Cx46 internalized or on the surface of the membrane. Images were processed using ImageJ. Scale bar 25 µm.

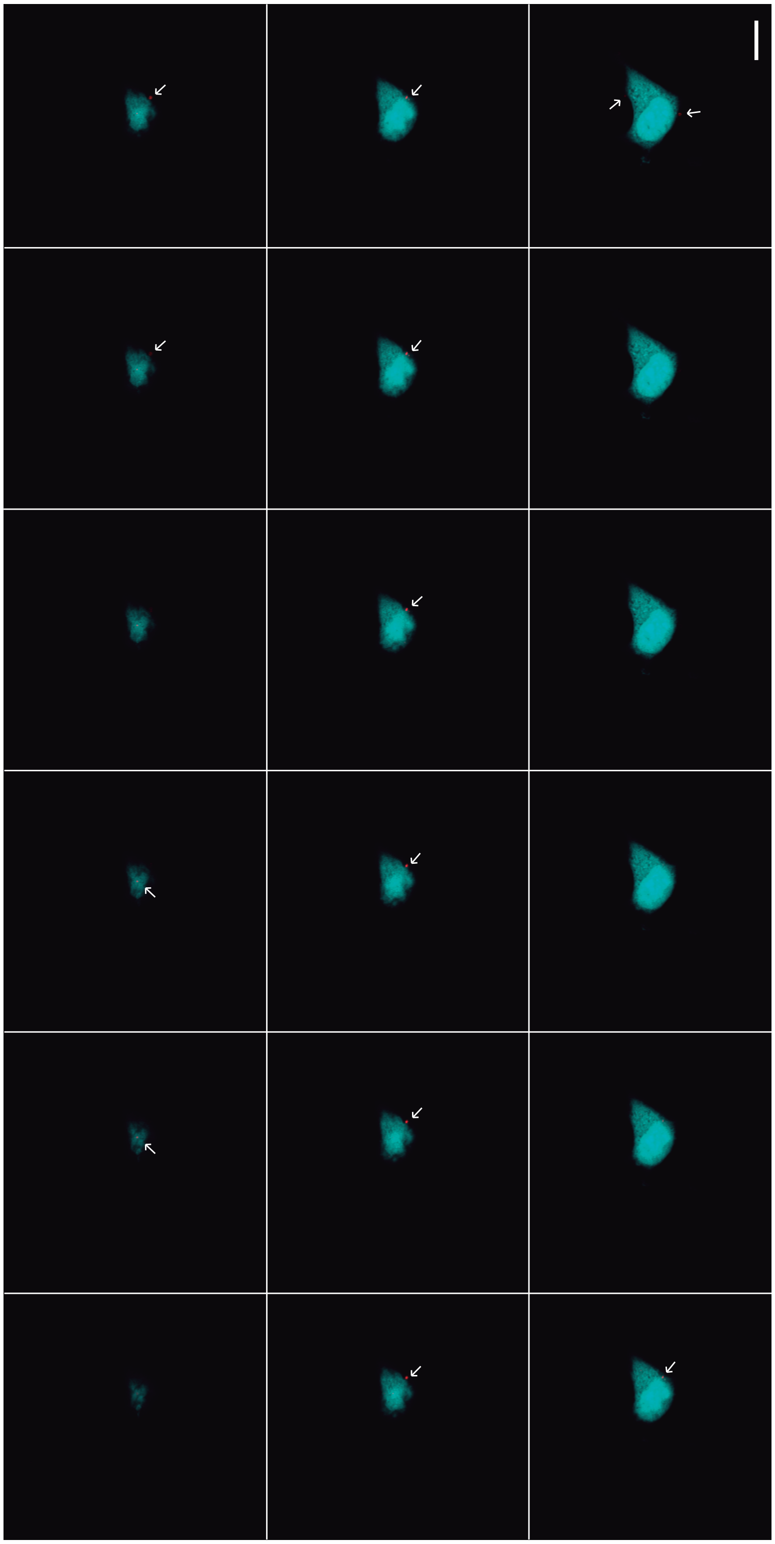

Supplementary figure 2. Confocal microscope analysis of EVs-Cx46 internalization in MCF-7Cx46-GFP and MCF-7 cells at 3 hours. Z-stack images were collected at 0,5 μm intervals. EVs-Cx46 (red) and Plasma membrane (cian). White arrows indicate the EVs-Cx46 internalized or on the surface of the membrane. Images were processed using ImageJ. Scale bar 25 μm.
